# Case report of multiple mesenteric mycotic aneurysms after perforated appendicitis

**DOI:** 10.1016/j.ijscr.2021.01.064

**Published:** 2021-01-20

**Authors:** Anne C.M. Cuijpers, Sanne W. de Boer, Christiaan van der Leij, Marielle M.E. Coolsen

**Affiliations:** a Department of Surgery, Maastricht University Medical Centre, PO Box 5800, 6202 AZ Maastricht, the Netherlands; b Department of Radiology, Maastricht University Medical Centre, PO Box 5800, 6202 AZ Maastricht, the Netherlands

**Keywords:** CRP, C-reactive protein, CT, computed tomography, SCARE, Surgical CAse REport, Mycotic aneurysm, Appendicitis, Endovascular, Embolization, Case report

## Abstract

**Introduction and importance:**

Mycotic aneurysms are a severe and sometimes life-threatening complication of infections or sepsis. However, mycotic aneurysms of mesenteric arteries caused by a purulent peritonitis following perforated appendicitis are very rare and not previously reported. This case report contributes to the awareness and treatment of this rare complication.

**Case presentation:**

We present a case of a middle aged patient with a purulent peritonitis after perforated appendicitis. Postoperatively, recovery was not as expected. One week after laparoscopic appendectomy, CRP increased and haemoglobin levels dropped. Abdominal CT imaging revealed several mesenteric mycotic aneurysms. Because of aneurysmatic rupture, coiling of several mesenteric arteries was performed, despite the risk of bowel ischemia. After long-term antibacterial and antifungal treatment, the patient recovered completely.

**Clinical discussion:**

Formation of mycotic aneurysms in mesenteric arteries is very rare and optimal treatment options are debatable. Given the high mortality of conservative therapy with antibiotics alone, treatment of mycotic aneurysms by surgery or endovascular treatment is strongly advised. In our case, it was decided to treat the aneurysms by endovascular coil embolization and to observe whether bowel ischemia would occur, because aneurysms were present in multiple mesenteric arteries.

**Conclusion:**

Mycotic aneurysms of several mesenteric arteries should be considered as a rare but potential complication of perforated appendicitis with purulent peritonitis. In case of rupture, successful treatment can be achieved by embolization followed by long-term antibiotics. A multidisciplinary approach including surgery, interventional radiology and microbiology is highly recommended and transfer to a tertiary referral centre should be considered.

## Highlights

• Mesenteric mycotic aneurysms after perforated appendicitis are extremely rare.
• Optimal treatment options are debatable.
• Successful treatment can be achieved by embolization and long-term antibiotics.

## 1 Introduction

Mycotic aneurysms are a well-known but severe complication of infections, sepsis or endocarditis, arising from vessel damage due to adjacent infections or spread of micro-organisms [1]. However, mycotic aneurysm formation caused by a perforated appendicitis is very rare, especially mycotic aneurysms of the mesenteric arteries. In previous literature, only a few comparable cases have been described reporting the formation of mycotic aneurysms of the abdominal aorta, iliac arteries and uterine arteries after perforated appendicitis [[2], [3], [4], [5], [6], [7]].

We present a case of a middle aged patient who developed several mesenteric mycotic aneurysms one week after undergoing laparoscopic appendectomy for perforated appendicitis with purulent peritonitis in our University Hospital. To our knowledge, this is the first description of such a case. The aim of this case report is to contribute to the awareness and treatment of this rare complication. This case report has been reported in line with the Surgical CAse REport (SCARE) 2020 criteria [8].

## 2 Presentation of case

**Fig. 1:**
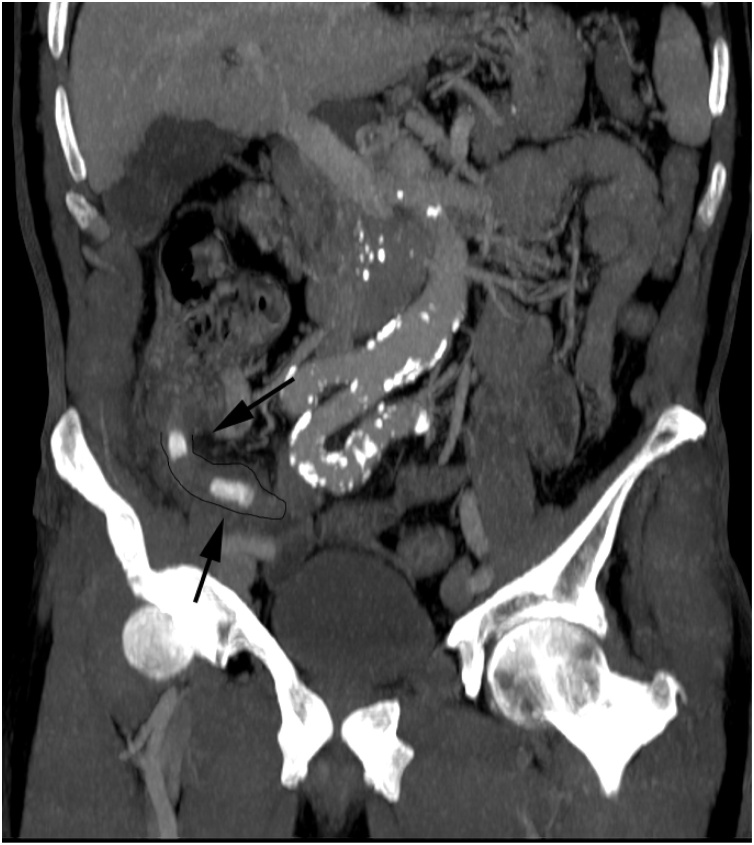
CT scan showing perforated appendix containing two appendicoliths.

A 69-year old male patient was admitted to the emergency department. His medical history included hypertension and prostate cancer. Treatment of hypertension consisted of lisinopril 10 mg once daily and metoprolol 50 mg once daily. Furthermore, the patient was on a simvastatin 40 mg once daily. The prostate cancer was treated by radiotherapy and hormone therapy and the patient was currently under active surveillance. He quit smoking six months ago and reported no allergies. The patient presented with continuous right-sided lower abdominal pain and signs of peritonitis. Laboratory results showed signs of infection with leucocytosis of 16.2 × 10^9/L and C-reactive protein (CRP) of 231 mg/L. Abdominal ultrasound and computed tomography (CT) scan revealed a perforated appendix containing two dense structures that were considered appendicoliths and two small abscesses (Fig. 1). The abscesses were considered too small for percutaneous drainage. Intravenous amoxicillin/clavulanic acid (1000/200 mg four times daily) was started and the patient was taken into the operating theatre where a laparoscopic appendectomy was performed.

The procedure was performed by a senior resident with five years of experience. Intraoperative findings confirmed a perforated appendicitis with a purulent peritonitis. During the procedure a free appendicolith was noted and removed. The appendix was stapled of the caecum at its base using an Endo GIA™ stapling device with a 45 mm Purple cartridge (Medtronic). After removal of the specimen and local irrigation with saline solution no further contamination was noted. Before closure, the omentum was placed in the right lower quadrant. No complications occurred during the procedure.

**Fig. 2:**
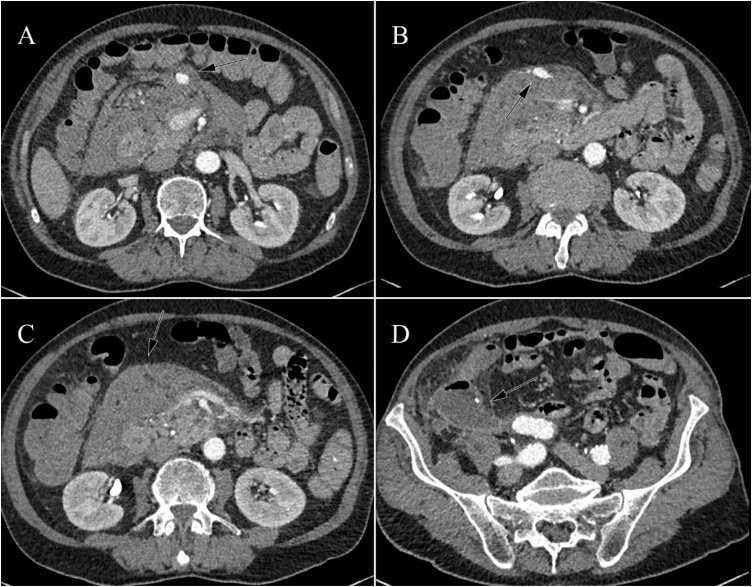
CT angiography scan showing multiple aneurysmatic dilatations (A and B), a panniculitis like infiltrating mass (C) and a new abscess in the right lower abdomen (D).

Postoperatively, the patient did not recover as expected with a slow decline in leucocyte count and CRP-levels. At day 8 postoperatively, CRP increased and haemoglobin levels dropped from 14.5 g/dL to 11.28 g/dL. A CT angiography scan was performed revealing a new abscess in the right lower abdomen and multiple aneurysmatic dilatations arising from the medial colic artery, pancreaticoduodenal artery, multiple intrahepatic arteries and the splenic artery surrounded by a large infiltrating mass (Fig. 2). Aneurysmatic rupture with the formation of a large haematoma, acute pancreatitis and mesenteric panniculitis triggered by peritonitis and recent surgery were considered as differential diagnoses. On the CT scan that was performed at admission no aneurysmatic dilatations as well as no signs of bleeding, pancreatitis or panniculitis like abnormalities were visualized. A low lipase level excluded pancreatitis and mesenteric panniculitis was considered unlikely because it is a non-invasive disorder not causing vascular involvement and therefore no explanation for the aneurysm formation [9]. Formation of multiple mycotic aneurysms due to the purulent appendicitis accompanied by aneurysmatic rupture was strongly suspected based on the sudden drop in haemoglobin and the large infiltrating mass surrounding the aneurysms and therefore treated as such.

**Fig. 3:**
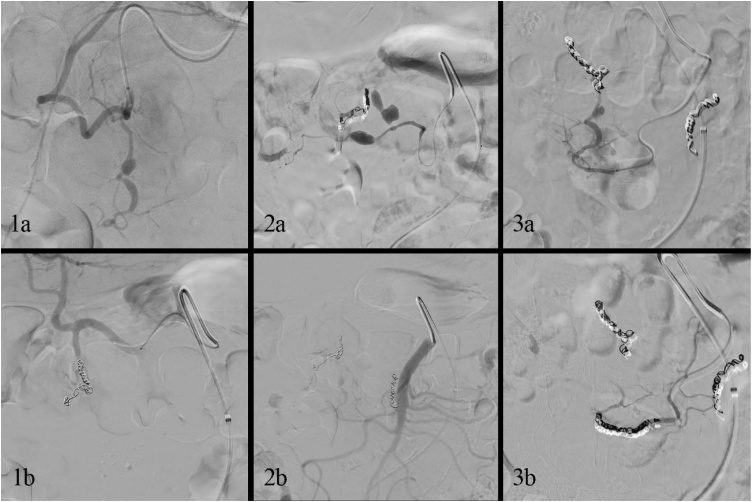
Angiography showing aneurysmatic dilatations of the gastroduodenal (1a), medial colic (2a), and inferior pancreaticoduodenal artery (3a), and successful coil embolization of these arteries in 1b, 2b and 3b respectively.

An endovascular coiling of the gastroduodenal, inferior pancreaticoduodenal and medial colic artery was performed by two experienced interventional radiologists, despite the risk of bowel ischemia (Fig. 3). Subsequently, the abscess was drained percutaneously. Amoxicillin/clavulanic acid was replaced by piperacillin/tazobactam (4000/500 mg three times daily) and anidulafungin (100 mg twice daily). After 24 h of monitoring in the intensive care unit, haemoglobin levels remained stable, no bowel ischemia occurred and the patient was transferred to the surgical ward.

Bacterial cultures revealed *Haemophilus parainfluenzae* and *Bacteroides fragilis* bacteria, both sensitive to piperacillin/tazobactam. Other organisms predominantly found to produce mycotic aneurysms like *Staphylococcus aureus* and *Salmonella* species were excluded.

Other sites of infection and endocarditis were ruled out by positron emission tomography CT and trans-oesophageal ultrasound. The patient recovered slowly and was discharged three weeks after initial presentation. He continued intravenous ceftriaxone (1000 mg twice daily) and oral metronidazole (500 mg three times daily) at home for a total of six weeks. He returned to the outpatient clinic for several follow up assessments with the last visit one month after antibiotic cessation. Inflammation parameters returned to normal and the patient recovered completely. The patient was contacted one year later and he remained without complaints. The coil embolization was experienced as unpleasant and lengthy, but the patient was pleased with the aftercare and eventual outcome.

## 3 Discussion

Mycotic aneurysms are well-known complications of septic episodes and infectious diseases and are associated with a significant morbidity and mortality, especially in case of severe sepsis or arterial rupture [1]. However, purulent peritonitis due to a perforated appendicitis as a cause of mycotic aneurysm formation is rare and only described in a few cases [[2], [3], [4], [5], [6], [7]]. Arteries can be affected by bacterial or fungal organisms from a neighbouring source of infection, causing aneurysmatic dilatation and possibly rupture of the vessel wall. Other causes of mycotic aneurysms are haematogenous spread of micro-organisms as in sepsis or bacterial endocarditis [1]. Gram-positive cocci, *Salmonella* species and fungi like *Candida albicans* and *Aspergillus* are the most commonly found organisms [1,10]. However, also gram-negative bacteria and *Bacteroides fragilis*, as was the case in our patient, have been described [10].

Treatment of mycotic aneurysms is based on long-term antibiotics and exclusion of the aneurysm, given the high mortality of conservative therapy with antibiotics alone. Solutions to exclude the aneurysm consist of surgical ligation, open arterial reconstruction or endovascular techniques by either covered stenting or coiling [1,11]. Formation of mycotic aneurysms in mesenteric arteries is very rare and optimal treatment options are debatable. Kordzadeh et al. reported that mesenteric aneurysmectomy alone was associated with significant bowel resection, except in patients with well-established collateral arteries. Furthermore, the use of endovascular stents is limited because of the risk of further colonization and possible technical difficulties based on the location of the aneurysm [11]. In the present case, both endovascular and open vascular reconstruction options were considered. After multidisciplinary meeting between surgeons, vascular surgeons and interventional radiologists, it was decided to exclude the aneurysms with endovascular coil embolization and to observe whether bowel ischemia would occur. It was opted not to perform primary bowel resection because, besides the medial colic artery, aneurysms were also present in other mesenteric arteries. Optimal antibiotic treatment was determined in consultation with the microbiology department.

## 4 Conclusion

The clinical lessons that can be learned from this case are that mycotic aneurysms of several mesenteric arteries caused by a perforated appendicitis with purulent peritonitis are a rare phenomenon but should be considered as a possible complication. In case of aneurysmatic rupture, a timely and accurate diagnosis is needed for proper treatment and good clinical outcomes. Successful treatment can be achieved by coil embolization followed by long-term antibiotics and antifungal treatment. Multidisciplinary collaboration between the surgical, interventional radiology and microbiology department is highly recommended. Furthermore, transferring patients to a tertiary referral centre with an adequate level of facilities and expertise should be considered.

## Declaration of Competing Interest

The authors declare that they have no conflict of interest.

## Funding

This research did not receive any funding.

This research did not receive any specific grant from funding agencies in the public, commercial, or not-for-profit sectors.

## Ethical approval

This research is exempt from ethical approval as this is not a research study.

## Consent

“Written informed consent was obtained from the patient for publication of this case report and accompanying images. A copy of the written consent is available for review by the Editor-in-Chief of this journal on request”.

## Author contribution

Anne Cuijpers: Investigation, Writing – Original Draft, Visualization.

Christiaan van der Leij: Review & Editing.

Sanne de Boer: Review & Editing, Visualization.

Mariëlle Coolsen: Writing – Review & Editing.

## Registration of research studies

Not applicable.

## Guarantor

All authors, Drs. Anne C.M. Cuijpers, Dr. Sanne W. de Boer, Dr. Christiaan van der Leij and Dr. Marielle M.E. Coolsen have full access to the data and were involved in the decision to publish. All authors accept full responsibility for the work and the conduct of the study.

## Provenance and peer review

Not commissioned, externally peer-reviewed.

## References

[1] Lee W.K., Mossop P.J., Little A.F., Fitt G.J., Vrazas J.I., Hoang J.K. (2008). Infected (mycotic) aneurysms: spectrum of imaging appearances and management. Radiographics.

[2] Chandler B.T., Ryer E.J., Keyser B.M., Elmore J.R. (2017). A hybrid approach to appendicitis with right external iliac artery pseudo aneurysm: a case report. Int. J. Surg. Case Rep..

[3] Garb M. (1994). Appendicitis: an unusual cause of infected abdominal aortic aneurysm. Australas. Radiol..

[4] Hsu J.S., Wu I.H., Liu K.L. (2012). A common disease with an unusual complication of acute abdomen. Gastroenterology.

[5] Jewkes A.J., Black J. (1989). Infection of an abdominal aortic aneurysm from an appendix abscess. J. Cardiovasc. Surg. (Torino).

[6] Polat K.Y., Aydinli B., Keles M., Uyanik A., Ozturk G., Ceviz M. (2011). Spontaneous mycotic external iliac artery aneurysm rupture after perforated acute appendicitis in a renal allograft recipient. Exp. Clin. Transplant..

[7] van Doorn D.E.A., van Leuken M., Rijnders R.J.P. (2014). Embolization of a left uterine artery mycotic aneurysm after a neglected, perforated appendicitis before delivery. Clin. Case Rep..

[8] Agha R.A., Franchi T., Sohrabi C., Mathew G., Kerwan A., Thoma A. (2020). The SCARE 2020 guideline: updating consensus Surgical CAse REport (SCARE) guidelines. Int. J. Surg..

[9] Daskalogiannaki M., Voloudaki A., Prassopoulos P., Magkanas E., Stefanaki K., Apostolaki E. (2000). CT evaluation of mesenteric panniculitis: prevalence and associated diseases. AJR Am. J. Roentgenol..

[10] Pirvu A., Bouchet C., Garibotti F.M., Haupert S., Sessa C. (2013). Mycotic aneurysm of the internal carotid artery. Ann. Vasc. Surg..

[11] Kordzadeh A., Watson J., Panayiotopolous Y.P. (2016). Mycotic aneurysm of the superior and inferior mesenteric artery. J. Vasc. Surg..

